# Does Covert Retrieval Benefit Adolescents’ Learning in 8th Grade Science Classes?

**DOI:** 10.3390/bs15070843

**Published:** 2025-06-23

**Authors:** Amber E. Witherby, Paige E. Northern, Sarah K. Tauber

**Affiliations:** 1 Department of Psychological Sciences, Creighton University, Omaha, NE 68178, USA; amberwitherby@creighton.edu; 2 Department of Psychology and Counseling, Southeast Missouri State University, Cape Girardeau, MO 63701, USA; penorthern@semo.edu; 3 Department of Psychology, Texas Christian University, Fort Worth, TX 76129, USA

**Keywords:** covert retrieval, overt retrieval, adolescents, STEM education

## Abstract

Retrieval practice can benefit students’ long-term learning. However, no prior investigations have explored the degree to which response mode—overt versus covert retrieval—moderates the impact of retrieval practice on adolescents’ learning in a classroom context. To explore this issue, 8th grade students learned science concepts that were required for their class. They learned terms in their middle school classrooms by recalling and writing them down (overt retrieval), mentally recalling (covert retrieval), or restudying definitions. They practiced each strategy in 5 sessions and took final tests 2 days later. The impact of covert retrieval on students’ learning was similar to that of restudy, and both covert retrieval and restudy were less beneficial relative to overt retrieval. Treatment package effectiveness differed somewhat between students and terms. These outcomes are generally consistent with the retrieval dynamics hypothesis and reveal that response mode can impact the effectiveness of retrieval practice in middle school classrooms.

## 1. Introduction

Retrieval practice involves bringing concepts to mind from memory, and it can support students’ long-term retention of information (Roediger & Karpicke, 2006a; for reviews, see Adesope et al., 2017; Carpenter et al., 2022; McDermott, 2021; Rowland, 2014). For instance, students have shown enhanced learning of STEM concepts following retrieval practice (e.g., Darabi et al., 2007; Kang et al., 2011; Pan et al., 2016, 2019), and retrieval practice can benefit learning in educational contexts (McDaniel et al., 2007; for reviews, see Agarwal et al., 2021; Moreira et al., 2019). Most of these investigations focused on learning in higher education. Even so, retrieval practice can also enhance adolescents’ learning (for reviews, see Agarwal et al., 2012; Roediger & Karpicke, 2006b). However, *how* students engage in retrieval may be critical, and no research has evaluated if covert (mental) retrieval is effective for adolescents’ long-term learning. Our goal was to investigate this issue by recruiting 8th grade science students, many of whom were from underrepresented groups.

Retrieval practice can be beneficial for middle school students’ learning (Coyne et al., 2015; Fritz et al., 2007; McDaniel et al., 2011, 2013; McDermott et al., 2014; Roediger et al., 2011). For instance, McDaniel et al. (2011) had 8th grade science students take low-stakes quizzes to encourage retrieval practice for facts from the class content. Students’ exam performance was better for facts that appeared on the quizzes relative to facts that did not. Adolescents also self-report that retrieval practice reduces test anxiety and helps them learn (Agarwal et al., 2014). However, students can engage in retrieval practice in a variety of ways, and the mode of retrieval may moderate the effectiveness of this study strategy.

Most retrieval practice research requires that students generate a response by typing, writing, or speaking their answers. This overt retrieval approach is logical because researchers can evaluate the quality of students’ responses during learning, and it simulates some practices used by students. For instance, during group study, students can quiz each other on class concepts and respond aloud. College students also report responding to questions in class when their teachers use iClickers (Witherby & Tauber, 2019). However, students do not always generate an overt response when recalling concepts during study or answering questions during class. Instead, they recall information from memory without producing an explicit response, which is referred to as covert retrieval. When asked how students decide if they have correctly answered a question during study, few college students report having a partner check their responses or writing down answers correctly, both of which require an overt response (Wissman et al., 2011). As well, when a teacher asks a question of the class, often one or two students provide an overt response while the rest of the class mentally recalls their answers. As a final example, when studying by oneself in a quiet environment such as a library, students likely mentally retrieve answers to avoid disrupting others. Consistent with these examples, college students self-report being highly likely to use both covert and overt retrieval when studying (Witherby et al., 2025). Thus, it is important to evaluate the degree to which covert retrieval supports students’ long-term learning in educational contexts.

Mixed outcomes have been found for the impact of covert retrieval on college-aged students’ learning. Laboratory research has revealed response mode (overt versus covert) during retrieval to have little impact on learning (Higham et al., 2023; Putnam & Roediger, 2013; Smith et al., 2013). Smith et al. (2013) had students learn lists of words by category (e.g., types of vegetables) via overt retrieval, covert retrieval, no retrieval (or restudy), or restudy. A mini meta-analysis including their outcomes and others with similar methodology (Putnam & Roediger, 2013) showed retrieval practice to have a substantial impact on students’ learning (*d* = 1.10) when compared with restudy or no retrieval conditions. By contrast, mode of retrieval—overt versus covert—did not differentially impact students’ learning (*d* = −0.0027). Expanding on these outcomes, Sundqvist et al. (2017) reported a meta-analysis that included outcomes from Jönsson et al. (2014). They also found a low effect size (*d* = 0.07) comparing overt and covert retrieval, and thus concluded that overt and covert retrieval similarly impact learning.

Other evidence suggests that covert retrieval is inferior relative to overt retrieval for college-aged students’ learning (Jönsson et al., 2014; Abel & Roediger, 2018; Kubik et al., 2020; Tauber et al., 2018). Tauber et al. (2018) tasked college students with learning definitions for key terms. Students studied the key term definitions by practicing covert retrieval, overt retrieval, or restudying the information. Meta-analyses showed that covert retrieval was less beneficial for performance on a delayed test relative to overt retrieval, and covert retrieval did not differ from restudy. In a related literature, metacognitive researchers have found analogous outcomes (Ariel et al., 2021; Kubik et al., 2022; Tauber et al., 2015). In this work, overt retrieval practice is contrasted with restudy and conditions for which covert retrieval is presumed to occur. Under covert retrieval conditions, participants make judgments of learning (JOLs) after a delay and with only a cue available (for a review, see Rhodes & Tauber, 2011). JOL conditions are typically less beneficial for retention on delayed tests relative to overt retrieval (but see Rhodes & Tauber, 2011).

Differences between covert and overt retrieval may be particularly pronounced in applied learning contexts. In the only study that has evaluated this possibility, Sumeracki and Castillo (2022) had college students learn information in a classroom context that was required for their course (Experiment 2) or not (Experiment 1). Students learned the material by using overt retrieval, covert retrieval, restudy, or no retrieval (i.e., control condition without additional exposure to the information). As expected, performance on delayed tests was higher following overt retrieval relative to restudy or no retrieval. By contrast, covert retrieval did not differ from restudy; however, performance did not differ between covert and overt retrieval practice. Extending these outcomes to adolescents in middle school is challenging. Middle school classrooms substantially differ from college classes (e.g., classroom management requirements), and given that middle school students presumably engage in covert retrieval during class and when studying on their own, it is important to evaluate their ability to use covert retrieval to support their learning.

Our research is guided by theory in student learning—specifically, the retrieval dynamics hypothesis (Tauber et al., 2018). From the retrieval dynamics hypothesis, the demand that students face during mental retrieval impacts the degree to which retrieval practice will enhance their learning. From this perspective, students are most likely to practice thorough retrieval for simple information or short answers. In these instances, response modality (overt vs. covert) may have no impact or only a minor impact on enhanced learning from retrieval practice. By contrast, when mentally retrieving complex concepts, students can end retrieval without bringing to mind all parts of the concepts, thereby reducing the impact of retrieval practice on learning. The retrieval dynamics hypothesis suggests that this is most likely to occur during covert retrieval practice due in part to poor indications of how much students have successfully retrieved during retrieval practice. We evaluated this possibility with adolescents by using complicated concepts in the experiment.

To preview the novel reported experiment, we investigated the degree to which covert retrieval enhances adolescents’ long-term learning. To explore adolescents’ learning in middle school classrooms, we had students learn terms and definitions for their science class. As important, we included three treatment packages that offer practice with covert retrieval, overt retrieval, or restudy to add to the growing literature on methods to enhance adolescents’ STEM education. The treatment packages required that students study the definitions of some terms and practice retrieval (overt or covert) for others. Students took short-answer and multiple-choice tests. Consistent with prior research, we expected the overt retrieval treatment package to lead to superior test performance relative to the restudy treatment package (Adesope et al., 2017; Carpenter et al., 2022; McDermott, 2021; Rowland, 2014). It was an open question as to the impact of the covert retrieval treatment package on adolescents’ learning in a classroom context; however, from the retrieval dynamics hypothesis and based on recent research (Jönsson et al., 2014; Abel & Roediger, 2018; Kubik et al., 2020; Tauber et al., 2018), we expected the covert retrieval treatment package to be less effective relative to the overt retrieval treatment package.

## 2. Materials and Methods

### 2.1. Educational Context

We conducted the experiment in classrooms at a public middle school. The middle school has a population of approximately 400 students, includes grades 6–8, and primarily serves historically underserved youth. According to a 2020–2021 Texas Education Agency School Report Card, 93.9% of the student body was economically disadvantaged. A total of 77.3% of the student population identified as Hispanic, 17.1% as African American, 4.2% as White, 0.5% as Asian, 0.7% as multiethnic, and 0.2% as American Indian. According to the 2021 Federal Report Card for the school, only 28% of 8th grade students performed at grade level or above in science by the end of the course on the State of Texas Assessments of Academic Readiness (STAAR; 49% approached grade level, 7% mastered grade level). This report identified the school as high poverty with multiple teacher challenges: 18.2% were inexperienced, 12.6% were teaching with emergency or provisional credentials, and 32.9% were teaching subjects for which they were not certified.

To conduct this experiment, we worked with one science teacher and her classes. The teacher identified as 33 years old, White, and as a woman. She was in her first year of teaching general science courses to 8th grade students at the middle school. She held a doctoral degree in Science Education and reported being 41–60% confident (on a categorical scale using 0–20%, 21–40%, 41–60%, 61–80%, and 81–100%) in her knowledge of strategies that are effective for student learning. The teacher and the principal of the school provided consent for the experiment to be conducted in students’ classrooms. As well, guardians provided consent (documentation provided in English or Spanish), and students assented to take part in the experiment. The experiment was approved both by the Texas Christian University IRB and the Fort Worth Independent School District. All students were provided with a $20 gift card to compensate them for their participation, as was the teacher ($100 gift card).

### 2.2. Design and Participants

Treatment package (i.e., overt retrieval, covert retrieval, and restudy) was manipulated using a within-participant design so that all students in all classes practiced all strategies. Ninety-four 8th grade students enrolled in one of four general science classes were invited to participate in the experiment. The sample size was based on the number of students enrolled in the available classes with the goal of obtaining an adequate number of observations to detect the within-participant effect of treatment package on final performance anticipating potential missing data across sessions. A group size of 21 was estimated using G*Power 3 (Faul et al., 2007). Of the 94 students, 24 did not return the guardian consent form, assent form, or both, and 5 students failed to complete the final tests. Thus, the final sample size for all analyses was 65 students (*n* = 12 in the 9 a.m. class, *n* = 15 in the 12 p.m. class, *n* = 18 in the 1 p.m. class, and *n* = 20 in the 2 p.m. class). This obtained sample size exceeded the sample size estimate (*N* = 28) from a power analysis that would be necessary to detect the medium-sized within-participant effect of treatment package (*f* = 0.25, power at 0.80, alpha of 0.05) that was consistent with our predictions (Faul et al., 2007).

Students completed an extensive demographic questionnaire. All students reported being in 8th grade and planning to attend high school. Most students (*n* = 55, 83.3%) reported planning to attend college (*n* = 1 only attending if accepted, *n* = 7 not planning on attending college, *n* = 3 unsure). One student reported a diagnosis of Autism Spectrum Disorder (*n* = 4 did not respond), and 8 reported a learning disability diagnosis (*n* = 6 did not respond). The reported disabilities were dyslexia (*n* = 4), attention deficit hyperactivity disorder (*n* = 2), both dyslexia and attention deficit hyperactivity disorder (*n* = 1), and obsessive compulsive disorder (*n* = 1). Many students (*n* = 31, 46.9%) were expecting to get a B in their general science class (*n* = 15 expecting an A, *n* = 5 expecting an A or B, *n* = 1 expecting an A, B, or C, *n* = 1 expecting an A or C, *n* = 1 expecting an B or C, *n* = 3 expecting a C, *n* = 8 did not respond). In terms of self-reported GPA, several students (*n* = 28, 42.4%) reported that they earn an equal number of As and Bs (*n* = 2 mostly As, *n* = 4 mostly Bs, *n* = 17 an equal number of Bs and Cs, *n* = 4 mostly Cs, *n* = 11 reported another grade combination).

Students reported that the primary language(s) spoken at home was English (*n* = 31, 46.9%), Spanish (*n* = 23, 34.8%), or both English and Spanish (*n* = 7, *n* = 5 who did not respond). Some students reported living with both parents (*n* = 25, 37.9%) or multiple adults (combinations of parent(s), aunt/uncle, cousin, roommate, niece, sister-in-law, and grandparent(s), *n* = 24, 36.4%). A total of 10 students reported living in a single-adult (mother or grandmother) household, and fewer (*n* = 6) reported living with a parent and step-parent (*n* = 1 who did not respond). Few students reported that their mother completed high school (*n* = 20, 30.3%) or earned a GED (*n* = 1, *n* = 15 did not know, *n* = 5 did not respond), and 6 of these students reported that their mother completed college (*n* = 6 did not know). Similarly, few students reported that their father completed high school (*n* = 15, 22.7%, *n* = 20 did not know, *n* = 6 did not respond), and 7 of these students reported that their father completed college (*n* = 2 did not know).

On average, the students were 13.96 years old (*SD* = 1.11). Forty-seven percent identified as a girl and 51.5% as a boy (1 participant did not respond). Regarding ethnicity, 68.2% identified as Hispanic, 18.2% as Black, 9.1% as Multiethnic, 3% as Asian or Pacific Islander, and 1.5% as White. These demographic characteristics did not differ between the four science classes (*F* < 1, *p* = 0.826, χ^2^s ≤ 6.72, *p*s ≥ 0.348) (see online supplement for demographic data for each class—https://osf.io/p4cq8, accessed on 16 June 2025).

### 2.3. Materials

We collaborated with the teacher to create all experimental materials. We targeted concepts that students needed to master for their science class and for their state exams. We selected 15 earth science terms (e.g., *jet stream*) and definitions (*a narrow band of strong, fast winds high in the upper troposphere*) from the students’ middle school science textbook that were important for understanding the functioning of Earth’s oceans and atmospheres, as well as for illustrating the principles of energy and matter and of cause-and-effect (e.g., *meteorology, convection*; all materials provided in the online supplement). The research team drafted a definition of each concept, and the teacher evaluated them for correctness, meeting the goals of course, and test relevancy. As well, the teacher verified that the information had not yet been taught and was presented using language that was suitable for the students’ comprehension capabilities. All materials are available in the online supplement (https://osf.io/p4cq8, accessed on 16 June 2025).

### 2.4. Procedure

**Figure 1:**
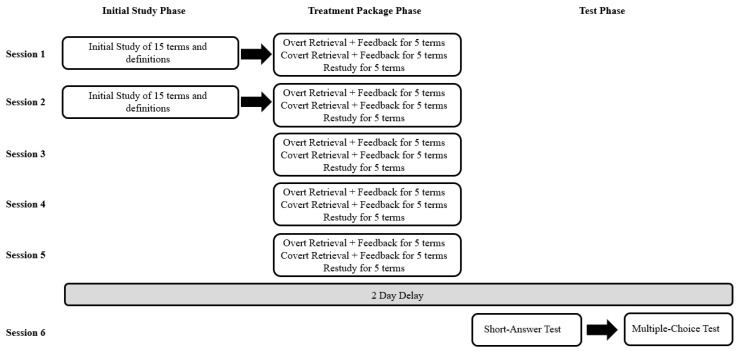
Overview of all experimental sessions. The procedure for session 2 was identical to that of session 1. The initial study phase refers to the first time terms and definitions were studied in that session, which was eliminated after session 2. The treatment package phase was maintained for sessions 3–5, and the procedure for sessions 3–5 was identical. Following a 2-day delay, during session 6, all students completed the final tests and demographic form.

The experiment consisted of 6 sessions (see Figure 1), each on a different day during students’ regularly scheduled class times at their middle school. Five sessions were on consecutive school days (i.e., Monday–Friday), and the final 6th session was on the following Monday (i.e., after a 2-day delay). Students participated in each session as a group with their classmates. Thus, students in the same class saw the same items in the same order and performed the same learning tasks per session. Even so, tasks were completed individually and without input from peers. During each session, 2 to 5 researchers were present who circulated around the room to ensure that students followed instructions, paid attention, and questions were answered.

For all sessions, instructions were provided on PowerPoint slides, they were read aloud, and time was provided to ask questions. Similarly, all to-be-learned materials were presented on PowerPoint slides and were read aloud by a researcher. In session 1, students were provided with a bird’s eye view of the activities. Students were then informed that they would study 15 terms and definitions, and they would practice learning them in three different ways. They were told that they would take a practice test and write the definition for some terms, take a practice test and silently think of the answer for some terms, and restudy the definitions for other terms. Students then received the following instructions:

“We selected **15 science terms** that you need to learn for your science class. Specifically, you will learn terms that are important for understanding Earth’s oceans and atmosphere. They are also important for learning about energy and matter as well as cause and effect.

Your task is to **learn the definition** to each term so that you will be able to remember it on a future memory test. The test will be short-answer format, which means that you will be given the terms and will be asked to write the definition to each.

**It is very important that you try your hardest to learn each definition.** Remember, these are concepts that you need to learn for your class, so if you work hard at learning them now, you may benefit later! You will be given 15 s to study each definition, which we will present on the screen and read aloud. Do you have any questions before we begin?”

**Figure 2:**
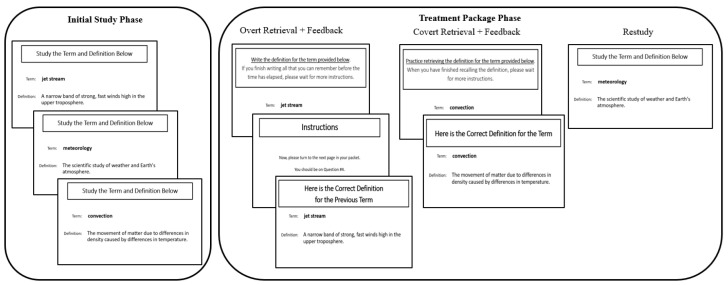
Initial study and treatment package procedures. The initial study phase involved studying 15 terms and their definitions. The treatment package phase involved studying the terms from the initial study phase in one of three ways: overt retrieval with feedback, covert retrieval, or restudy.

Next, using the time feature for slide transitions in PowerPoint, students studied each term and definition one at a time for 15 s (see Figure 2, left panel). Terms and definitions were presented in a random order for each class. After students studied the 15 terms and definitions, they began the treatment package phase (see Figure 2, right panel). Terms were randomly assigned to treatment package for session 1, and students continued to practice the terms using the same treatment package for all subsequent sessions. For instance, if the term *jet stream* was assigned to the overt retrieval treatment package, then all students in that class would practice overt retrieval for this concept for all sessions. We used the four classes to counterbalance the order of treatment packages.

Treatment package was blocked during learning and the order was counterbalanced across classes. We used this strategy to prioritize altering the order of treatment packages across classes and sessions to ensure that students were exposed to the treatment packages in different orders. This was critical due to the within-participant manipulation of treatment package. Put differently, our strategy ensured that conclusions drawn from the final test data were not dependent upon a specific order of treatment package. It should be noted, however, that the treatment order was imperfect given that data were collected from four classes, across 5 sessions, and 6 treatment package orders (see Table 1).

During each session, there was a block of 5 terms in the restudy treatment package, 5 terms in the overt retrieval treatment package, and 5 terms in the covert retrieval treatment package. The order of term and definition pairs within each block was randomized per session. To illustrate, convection was learned with the covert retrieval treatment package by the 12 students in the 9 a.m. class. In session 1, it was the 5th term in the third block (O,R,C order); in session 2, it was the 4th term in the second block (O,C,R order); in session 3, it was the 3rd term in the third block (R,O,C order); in session 4, it was the 1st term in the second block (R,C,O order); and in session 5, it was the 2nd term in the first block (C,O,R order). Thus, across classes, each term and definition were learned using each treatment package, with one exception. Due to experimenter error, the term meteorology was not assigned to the overt treatment package. A limitation of this approach is that the assignment of term and definition pairs to each treatment package across sessions was unbalanced (see Table 2). This occurred for two reasons. First, the number of students enrolled differed between classes (*n*s = 12, 15, 18, 20). Second, as noted above, the overt retrieval treatment package was never offered for one term (meteorology), which led to the restudy treatment package being offered in three classes. As a result, to-be-learned content was confounded with classes, a concern that we revisit in the General Discussion.

For the overt retrieval treatment package, students were given a handout with space to write the definition of the terms. Each term appeared on an individual page, and each page had space for students to write the definition for the corresponding term. Students received the following instructions:

“You are now going to take a **practice test on some of the terms and definitions**. To do so, you will practice retrieving the definition to each term in preparation for the final memory test.

You will be presented with each term one-at-a-time. For this test, your task is to **write down the definition to each on the paper provided**. You will be given 30 s to write each definition.

**IMPORTANT: You should do your best to recall and write down as much of the definition as you can.** Think about this like short answer questions on a test, in which you are asked to write down the whole definition for a term. The important thing is to **really try to recall and write down each definition.** When you have finished writing the definition for the term, we will provide you with **feedback as to the correct definition for it**, which we will provide on the screen and read aloud.”

Then, a term was presented, and students wrote its definition. Using the time feature for slide transitions in PowerPoint, students were allowed 30 s for overt retrieval, but it was immediately apparent that this did not give them adequate time to write the definition for each term. Thus, after the first session, the time for overt retrieval was extended to approximately 90 s per term for students in all classes. Following overt retrieval, students turned the page to prevent them from editing their responses, and they received feedback for the term. Specifically, the term and full definition were presented. Students turned in their handouts after they had written the definition and received feedback for the 5 terms assigned to overt retrieval practice.

Unlike the overt retrieval treatment, students did not write down their responses when presented with term in the absence of the definition for the covert retrieval treatment. For the covert retrieval treatment package, consistent with prior research (e.g., Tauber et al., 2018) students received the following instructions:

“You are going to take a **practice test on some of the terms and definitions**. To do so, you will practice retrieving the definition to each term in preparation for the final memory test.

You will be presented with each term one-at-a-time. For this test, your task is to **silently retrieve the definition to each term**. You will be given 30 s to think of the definition for each.

**IMPORTANT: You should do your best to recall as much of the definitions as you can.** Think about this as if you and a friend were quizzing each other on terms for an upcoming test in your class—if your friend gave you a term, you’d try to come up with the entire definition. Or, think about it like a practice test, in which you were asked to retrieve the entire definition for each term. In both cases, you would try to retrieve the entire definition for each term. That is what we’d like you to do on each trial here.

The important thing is to **really try to recall each definition**. On each trial, you should not just read the term and assume that you know it because it seems familiar. Rather, use the term as a cue to try to silently recall the definition from memory. When you have finished retrieving the definition for the term, we will provide you with **feedback as to the correct definition for it**, which we will provide on the screen and read aloud.

Remember, you are learning the definitions for each term in preparation for a future memory test. On that test you will be provided with the terms, and you will be required to write the full and correct definition for each. Do you have any questions before we begin?”

Each term was then presented alone for 30 s, which was ensured by using the time feature for slide transitions in PowerPoint, and students used the time to covertly retrieve the definition for each term. The researchers asked the students if more time was needed for covert retrieval, and because no students expressed the need for additional time, the time remained the same across sessions. Students were not allowed to write anything during covert retrieval. Students received corrective feedback that was identical to that of overt retrieval.

For the restudy treatment package, students received the following instructions:

“You are going to **restudy some of the terms and definitions**. To do so, you will study each term and definition in preparation for the final memory test. The terms will be presented one-at-a-time on the screen and will be read aloud. You will be given 30 s to study each definition. Do you have any questions before we begin?”

The PowerPoint slide transitions were set so that each term and definition was presented alone for 30 s, and students used the time to study each. Students were not allowed to write anything during restudy. The researchers asked the students if more time was needed for restudy, and because no students expressed the need for additional time, the time remained the same across sessions. Thus, the restudy treatment package differed from both overt and covert retrieval because the term and definition were always presented together, which prevented students from needing to retrieve anything from memory to define them.

The session 2 procedure was identical to that of session 1. Specifically, students had an initial study phase of all 15 terms and their associated definitions, which were presented one at a time for 15 s each using PowerPoint slide transitions. The order of the items was randomized anew. Then, students learned 5 terms and definitions with the covert retrieval treatment package, 5 terms and definitions with the overt retrieval treatment package, and 5 terms and definitions with the restudy treatment package. In sessions 3–5, the initial study phase was eliminated. Otherwise, the procedure was identical to that of sessions 1 and 2.

Session 6 occurred two days after session 5. Students received instructions that they would take a short-answer test, a multiple-choice test, and complete a demographic form. All tasks were self-paced. Students in each class received the same instructions and completed the tasks in the same order. The following instructions were given before beginning the short-answer test:

“First, you are going to take a **final short-answer test** over the terms and definitions that you have studied last week. For each question, your task is to write the full and correct definition for each term. So, please do your best to recall as much as you can for each question!

**Important:** you must do your best to **answer EVERY question** on this test. You cannot leave any blank. Once you have your paper, you are free to begin! If you have any questions, simply raise your hand and a researcher will come to you.”

On the short-answer test, students were given a paper packet with the 15 key terms with space for students to write the definition. The order of terms in the packet was randomized per class, and students could complete the test by writing definitions to the terms in any order desired (e.g., they could skip terms and then return to them). A researcher checked to make sure that students had attempted to define each term. If any definitions were left blank, the test was returned, and the student was encouraged to try their best to define all terms. No feedback was provided on the short-answer test. Students then received the following instructions for the multiple-choice test:

“Next, you are going to take a **final multiple-choice test** over the terms and definitions that you have studied last week. For each question, your task is to select the definition that best corresponds to the term.

**Important:** you must do your best to **answer EVERY question** on this test. You cannot leave any blank. Once you have your paper, you are free to begin! If you have any questions, simply raise your hand and a researcher will come to you.”

For the multiple-choice test, students were provided with a paper packet with multiple questions on each page. The test consisted of 15 four-alternative forced-choice questions (see materials posted online). Thus, for each question, a term was provided (e.g., *What is the Coriolis Effect?*) with four options from which to select. Similar to the short-answer test, for the multiple-choice test, questions order was randomized per class, and the options for each question were presented in the same fixed order for all students. Students could complete the test by answering the questions in any order (e.g., they could skip questions and return to them later). Feedback was not provided on the multiple-choice test. Researchers checked that all questions were answered before allowing the student to move to the next task. If there were questions that had not been answered, the test was returned to the student to complete. Last, students and the teacher completed a demographic form, were debriefed, and were compensated. Students were also provided with a debriefing statement (in English or Spanish) to bring home to their guardians.

The teacher reported that during the experiment (all 6 sessions) she did not do anything differently with her four classes that deviated from normal classroom activities. On 3 days for all classes, at least one term that was used in the experiment was used during class time outside of the context of the experiment. For one of these days, students completed a textbook reading that covered most of the terms being taught during the experiment. Students had no other assignments, tests, or class activities associated with the terms on Earth’s oceans and atmospheres.

### 2.5. Data Scoring

Each definition was composed of idea units. For example, the definition for the term *jet stream* contained 2 idea units: (1) *A narrow band of strong, fast winds* (2) *high in the upper troposphere*. The number of idea units ranged from 1 to 5 per definition (see the online supplement for the scoring rubric). Two independent raters scored all responses for overt retrieval practice from sessions 1–5 and for the short-answer test from session 6. For each idea unit, trained raters assigned 0 (no credit), 0.5 (partial credit), or 1 (full credit), depending on the completeness and correctness of each student’s response. The mean percent correct for each term was calculated separately for each student and for each rater. For instance, the response “*the movement of air in the upper troposphere*” received partial credit (0.5) for unit 1 and full credit (1) for unit 2 from both independent raters, resulting in 1.5 total credits out of the 2 total credits possible for this concept, resulting in 75% correct for the concept jet stream this participant. For 71.4% of responses (2345 total), the raters’ scores were in agreement. Interrater reliability was acceptable to high (Pearson’s *r* = 0.640 to *r* = 0.985) and significant in all instances (*ps* ≤ 0.001; see https://osf.io/p4cq8, accessed on 16 June 2025 for all correlations). Even so, a third independent rater viewed and resolved all conflicts. Thus, all responses were scored by either 2 or 3 independent raters. The resulting scores were then used to calculate the percentage of idea units correctly retrieved during practice and on the short-answer test by summing the idea units earned for each term separately for each participant, dividing by the by the total idea units possible for that term, and multiplying by 100. Aggregate averages were then created by summing the percentage of idea units for each term and dividing by the number of students who contributed responses on the short-answer test to create the average for each term. Aggregate averages were created using the same steps for performance during overt retrieval practice.

## 3. Results

The outcomes of primary interest were students’ performance on the final short-answer and multiple-choice tests, so they are presented first. Next, we present students’ performance for overt retrieval during practice sessions. Because class sample sizes were low (i.e., *n* = 12 to *n* = 20), our goal was not to evaluate between-classroom effects, and the power analysis was conducted for the within-participant manipulation rather than the between-participant class variable, we focused analyses on the within-participant manipulation of primary interest. Raw data and analyses by class can be found in the online supplement (https://osf.io/p4cq8, accessed on 16 June 2025).

### 3.1. Analytic Plan

We used the Bonferroni correction for all pairwise comparisons. The Bonferroni correction adjusts the observed *p*-value to account for multiple comparisons of the same data, which reduced the chance of committing a Type 1 error. We also report Bayes factors for all analyses (BF) (Krushcke, 2013), and we used JASP statistical software version 0.18.3 (JASP Team, 2024) to conduct all Bayes analyses (e.g., Faulkenberry et al., 2020; van Ravenzwaaij & Etz, 2021). We used a Cauchy prior distribution with the JZS prior (r = 0.707). In contrast to traditional null hypothesis significance tests, Bayes factors allow for the quantification of evidence in support of both the null and alternative hypotheses. BF_01_ is a ratio that indicates the degree to which the null hypothesis predicts the data better than the alternative hypothesis. A BF_01_ value of 1 indicates that the null and alternative hypothesis are equally probably. A BF_01_ value less than 1 provides evidence that more strongly favors the alternative hypothesis, whereas a BF_01_ value greater than 1 provides evidence that more strongly favors the null hypothesis. In the case of all non-significant analyses, we report BF_01_. For ease of interpretation, for all significant effects, we report BF_10_, which is simply the reciprocal of BF_01_, and it indicates the likelihood that the alternative hypothesis better predicts the data compared to the null hypothesis. BF_10_ is interpreted similarly to BF_01_ (i.e., BF_10_ = 1 indicates equal support for the null and alternative hypotheses; BF_10_ < 1 indicates stronger support for the null over the alternative hypothesis; BF_10_ > 1 indicates stronger support for the alternative over the null hypothesis).

### 3.2. Percent Correct on the Final Short-Answer Test

**Figure 3:**
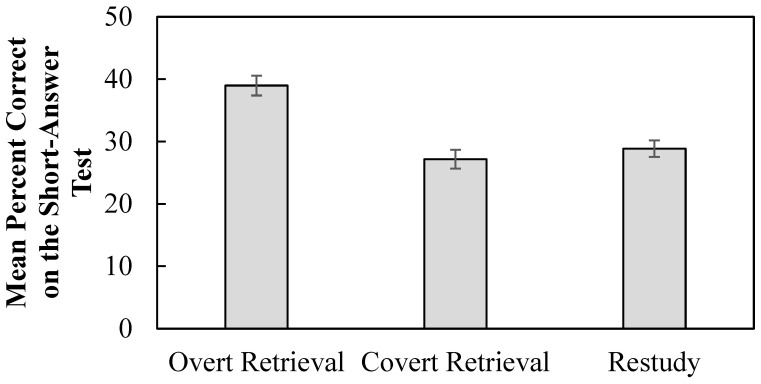
Mean percent correct on the short-answer test. Error bars reflect within-participant standard error of the mean (Loftus & Masson, 1994).

Students’ performance on the short-answer test tended to benefit most from the overt retrieval treatment package, and the impact of the covert retrieval treatment was similar to that of the restudy treatment (see Figure 3). In support of these conclusions, a 3-level (treatment package: overt, covert, or restudy) within-participant effect analysis of variance (ANOVA) was conducted on the percent correct on the final short-answer test. The analysis revealed that treatment package significantly impacted the percent correct on the final short-answer test, *F*(2, 126) = 12.76, *p* < 0.001, η_p_^2^ = 0.17, BF_10_ = 1960.10. The overt retrieval treatment (*M* = 39.19, *SE* = 2.88) led to significantly higher performance on the short-answer test relative to the covert retrieval treatment (*M* = 27.16, *SE* = 2.44) (*p* < 0.001, *d* = 0.56, BF_10_ = 354.65) and restudy treatment (*M* = 29.4, *SE* = 2.17) (*p* < 0.001, *d* = 0.50, BF_10_ = 161.08). There was no significant difference between the covert and restudy treatments on the short-answer test (*p* = 0.427, BF_01_ = 5.38).

Due to materials being confounded with classes, we also calculated average performance at the idea-unit level of each term separately for each treatment package. The term meteorology was not included in this data treatment to avoid confounding the calculation due to missing values. Consistent with the primary analysis above, a 3-level (treatment package: overt, covert, or restudy) repeated measures ANOVA revealed that there was a significant main effect of treatment package (*F*(2,86) = 8.84, *p* < 0.001, η_p_^2^ = 0.17, BF_10_ = 78.26). The pairwise comparisons confirmed that recall was significantly higher in the overt retrieval treatment package (*M* = 0.35, *SE* = 0.03) than in the covert retrieval (*M* = 0.27, *SE* = 0.03) (*p* = 0.001, *d* = 0.35, BF_10_ = 223.91) and restudy (*M* = 0.27, *SE* = 0.03) (*p* = 0.001, *d* = 0.33, BF_10_ = 16.99) treatment packages. Recall did not differ between covert and restudy treatments (*p* = 1.00, BF_01_ = 6.12).

For exploratory purposes, we also report analyses at the item level, given that the terms were unbalanced across treatment packages. The mean proportion correct per term and treatment package is reported in Table 3.

### 3.3. Percent Correct on the Final Multiple-Choice Test

**Figure 4:**
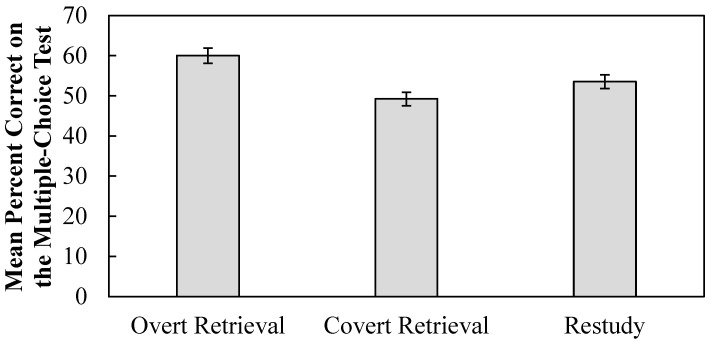
Mean percent correct on the multiple-choice test. Error bars reflect within-participant standard error of the mean (Loftus & Masson, 1994).

As evident from Figure 4, the overt retrieval treatment was associated with increased performance on the final multiple-choice test, whereas the covert retrieval treatment was not. In support of this conclusion, a 3-level (treatment package: overt, covert, or restudy) within-participant ANOVA was conducted on the percent correct on the multiple-choice test. Treatment package significantly impacted performance on the multiple-choice test (*F*(2, 122) = 4.86, *p* = 0.009, η_p_^2^ = 0.07, BF_10_ = 1.69). Performance was significantly higher for definitions that received the overt retrieval treatment (*M* = 0.62, *SE* = 0.04) relative to definitions that received the covert retrieval treatment (*M* = 0.50, *SE* = 0.03) (*p* = 0.007, *d* = 0.38, BF_10_ = 4.19). However, there was no significant difference in performance between definitions that received the overt retrieval and restudy treatments (*M* = 0.55, *SE* = 0.03) (*p* = 0.17, BF_01_ = 1.99).

We also explored multiple-choice outcomes at the item level. The mean proportion correct per term and treatment package is reported in Table 4. We computed a repeated-measures ANOVA on these means to evaluate whether treatment package affected multiple choice performance at the item level. The term meteorology was not included in this analysis to avoid confounding the calculation due to missing values. The outcomes revealed a non-significant effect of treatment package (*F*(2, 26) = 1.95, *p* = 0.169, η_p_^2^ = 0.13, BF_01_ = 1.56). Even so, one limitation of this analysis is that it was underpowered given that there are only 14 data points per treatment package. Consistent with this, the outcome of the Baye’s analysis did not provide strong evidence for or against either the null or alternative hypothesis. Numerically, the outcomes were consistent with the above analysis such that performance was higher for the overt retrieval treatment (*M* = 61.67, *SE* = 5.22) than for either the covert retrieval treatment (*M* = 51.48, *SE* = 4.83) or the restudy treatment (*M* = 54.48, *SE* = 5.52).

### 3.4. Percent Correct During Overt Retrieval Practice

**Figure 5:**
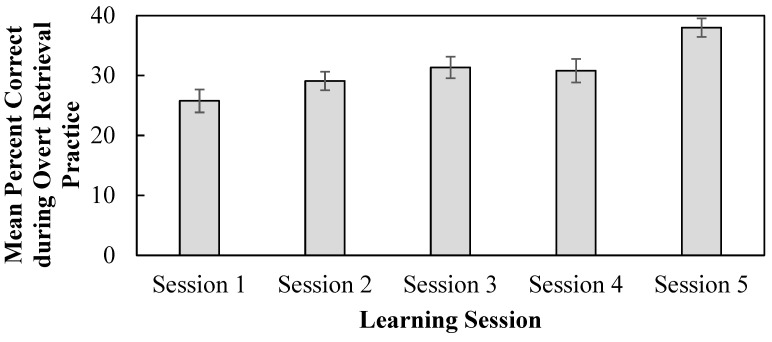
Mean percent correct during overt retrieval across learning sessions. Error bars reflect within-participant standard error of the mean (Loftus & Masson, 1994).

Percent correct during overt retrieval practice could only be assessed for terms and definitions assigned to the overt retrieval treatment because this was the only condition that required a response during learning. Students’ overt retrieval practice tended to improve across learning sessions (see Figure 5). In support of this conclusion, a 5-level (learning session: 1, 2, 3, 4, 5) within-participant ANOVA was conducted. Overt retrieval practice significantly differed between the sessions (*F*(4, 172) = 8.32, *p* < 0.001, η_p_^2^ = 0.16, BF_10_ = 1292.59). Session 5 overt retrieval practice was significantly higher relative to sessions 1–3 (*p*s ≤ 0.02, *d*s 0.28–0.54, BFs_10_ > 9.60). No other comparisons were significant (*p*s ≥ 0.07, BFs_01_ > 0.19).

## 4. Discussion

This experiment is the first to evaluate the impact of a covert retrieval treatment package on adolescents’ learning, and we explored this issue by using class concepts and evaluating students’ learning in middle school classrooms. Of primary interest, the covert retrieval treatment during multiple study sessions was less effective for adolescents’ long-term learning relative to the overt retrieval treatment. This inefficacy was found for both the short-answer and the multiple-choice tests, although specific terms may have contributed to the effect on the multiple-choice test. Consistent with these outcomes, students and teachers rate covert retrieval to be less effective than overt retrieval, and teachers indicate being less likely to recommend covert than overt retrieval to students (Witherby et al., 2025). Adolescents’ learning did not substantially differ depending on whether they received the covert retrieval or restudy treatment package. These outcomes are consistent with prior work demonstrating that response modality (covert versus overt retrieval) can moderate the impact of retrieval practice on students’ learning (Jönsson et al., 2014; Abel & Roediger, 2018; Kubik et al., 2020, 2022; Tauber et al., 2015, 2018; Ariel et al., 2021). As important, the reported outcomes advance the literature by establishing a treatment package effect for adolescents engaging in retrieval practice in educational contexts.

In contrast to the covert retrieval treatment, the overt retrieval treatment benefited students’ long-term learning. As expected, the overt retrieval treatment package led to higher average scores on the short-answer test relative to restudying the terms and definitions. Although the overt retrieval treatment did not statistically benefit students’ learning relative to the restudy treatment on the multiple-choice test, a small numerical advantage (overt retrieval treatment *M* = 0.62, *SE* = 0.04; restudy treatment *M* = 0.55, *SE* = 0.03) was observed. These outcomes are consistent with prior research (e.g., Darabi et al., 2007; Kang et al., 2011; Pan et al., 2016, 2019; for reviews, see Agarwal et al., 2012, 2021; Moreira et al., 2019; Roediger & Karpicke, 2006b).

Why can response mode impact the effectiveness of treatment package? There are likely two contributing factors. According to the retrieval dynamics hypothesis (Tauber et al., 2018), when learning information with multiple components, students face a high retrieval burden, which is not well supported by covert retrieval. Consider for instance that to correctly retrieve the definition for the term *cold front*, students must retrieve four complex units of information—(1) *the zone separating two air masses* (2) *of which the cooler, dense mass*, (3) *is advancing and replacing*, (4) *the warmer mass*. When covertly retrieving this definition from memory, students may halt retrieval prematurely due to familiarity with the concept or incorrect beliefs that information is well learned. By doing so, exhaustive retrieval is avoided, which decreases the effectiveness of retrieval practice. By contrast, because overt retrieval requires a written, typed, or spoken response, it is clearer when only partial (or no) information is generated. This likely encourages students to attempt full retrieval more often, which increases the effectiveness of retrieval practice. When the retrieval burden is low because students are learning less complex information, as used in some prior research, covert retrieval can be as effective as overt retrieval (Higham et al., 2023; Putnam & Roediger, 2013; Smith et al., 2013; Sundqvist et al., 2017). This is positive because covert retrieval can be relatively quick and easy to implement in classrooms. Teachers can construct retrieval practice questions on the fly and provide immediate feedback to their students. By contrast, methods to implement overt retrieval require more preparation for teachers (e.g., constructing quizzes before class) and can require additional course equipment (e.g., iClickers). Overt retrieval also requires that time be made available so that students can write their answers or say them out loud.

In addition to differences in the dynamics of retrieval between treatment packages, a second key factor to consider is the amount of time students spent on the learning task in each treatment package. The time during learning was held constant for the covert retrieval and restudy treatment packages and no students expressed needing more time to recall the definition or restudy it, which is consistent with college-aged students’ self-paced measures of reaction time during covert retrieval and restudy (Tauber et al., 2018). However, participants took longer to complete their overt retrieval responses relative to covertly retrieving definitions or restudying them. This unequal study time between treatment packages is important for overt retrieval outcomes. Specifically, it is likely that some portion of the benefit of the overt retrieval treatment package is attributable to increased time with the materials. This is a natural occurrence for the overt retrieval treatment package because it takes more time to generate a manual response relative to a mental response. As important, this experiment provides an index of the time needed by adolescents to use retrieval practice strategies in real educational settings. We think it unlikely that exposure time alone drives the benefits of the overt retrieval treatment because ample prior work has established that the quality of the learning strategy employed can overshadow simple exposure time.

Our outcomes suggest that response mode (overt vs. covert retrieval) impacts the effectiveness of students’ learning due to the dynamics of retrieval and the amount of time necessary to complete learning the learning task. The amount of time required to practice retrieval will depend on the response mode and complexity of the to-be-learned materials, so an important direction for future research will be to disentangle response mode from study time. Doing so will be important for considering not only the effectiveness of each treatment package, but also their efficiency. Students recalled the most with overt retrieval, but overt retrieval also consumed more time relative to covert retrieval and restudy. Increasing exposure time for covert and restudy conditions to match the needed time to generate an overt response would be experimentally simple; however, implementing the learning strategies in this way is unrealistic for an actual classroom context, and this applied focus drove our experimental design. Additional restudy or covert retrieval time beyond what students report as necessary to engage in the task would presumably lead to mind wandering or off-task engagement for many students in a classroom context. Indeed, the classroom teacher and school principal both lamented this as a regular concern in middle school classrooms. An alternative could be to allow adolescents to self-pace their retrieval practice during learning, measure individual reaction times, and establish the relationship between retrieval practice time and final test performance for both covert and overt response modalities. This is a fruitful direction for future research because it can increase understanding of treatment efficiency—i.e., how much knowledge is gained relative to the amount of time needed to use each strategy during learning.

There are other practical concerns to consider about implementing covert retrieval in middle school classrooms. Covert retrieval does not provide information on the accuracy of students’ responses, which makes it challenging for teachers to identify gaps in knowledge and determine if concepts need to be revisited. Unexpected distractions can occur during classes that may be particularly problematic for covert retrieval. Informal notes taken by the research team indicated that several students had their heads down, eyes closed, or were looking around the room and away from the screen during covert retrieval and restudy. In some instances, these behaviors may reflect deep thinking and comprehensive retrieval of definitions, but in others, students may be disengaged. Additional research is needed to systematically probe students for engagement during covert retrieval. To combat disengagement, Sumeracki and Castillo (2022) proposed that teachers combine in-class cold calling with covert retrieval, and an important direction for future research will be to consider this and other approaches for increasing the effectiveness of covert retrieval for supporting adolescents’ learning in classroom environments. Another practical direction for future research will be to explore methods to increase the effectiveness of the covert retrieval treatment when learning complex materials. Covert retrieval may necessitate scaffolding for success such as reminding adolescents how many components each definition has (e.g., “remember, the definition for this term has 4 key components and you should try to recall them all”) or teaching students strategies for updating their knowledge when feedback is provided (e.g., “locate all 4 components of the definition in the feedback. Which did you remember and which did you forget?”).

An important issue for both the educational context and experimental work is to measure student characteristics and explore the degree to which individual differences impact success with each treatment package. For instance, covert retrieval likely involves some ability to rely on an inner monologue, and some students may be better at this than others. Additionally, some students may need more time to engage in covert retrieval relative to others, and not all students will feel comfortable speaking up in class about needing more time to complete a task. Another factor that may be critical is students’ prior knowledge or level of expertise when learning new information using each treatment. Addressing these issues will be valuable for increasing understanding of how to best support students’ learning.

To contextualize our outcomes, we must note a few important limitations of the reported experiment. The biggest factor limiting aspects of our methodology was conducting this experiment in authentic adolescent science classrooms. Carrying out experimental work in a classroom context is difficult because there are many factors outside of the experimenters’ control. Examples include class enrollment numbers, non-random assignment to classes, class time availability, course schedule, and coordinating educational content with the science classes. These conditions make for a noisy assessment of students’ learning; however, carrying out this labor-intensive research is critical for investigating the degree to which laboratory effects generalize to a classroom context and a broad range of students (e.g., Agarwal et al., 2021; Schwieren et al., 2017). This step is critical for establishing best recommendations for educators, but reduced methodological control can make it challenging to draw firm conclusions from obtained classroom outcomes.

Given the classroom nature of this work, a limitation of our research is that counterbalancing of treatment packages and terms across classes and sessions was unbalanced and therefore terms were confounded with classes. This was driven by variance in class sizes, having access to only four science classes, and for one term, experimenter error (see procedure for details). Relevant to this issue, even though the course teacher verified that students had not been taught the STEM materials used in our experiment, we did not measure students’ prior knowledge of the concepts. Reported outcomes should be interpreted as directly relevant to the materials used with the degree to which they generalize to other materials as an open question for subsequent work. We recommend laboratory research to replicate this applied experiment adopting better counterbalancing as well as classroom research using the same materials as well as other content adolescents are required to learn in their STEM classes. With all these approaches, it will be important to quantify and evaluate the impact of characteristics of the to-be-learned materials (e.g., difficulty, complexity, abstractness) on learning.

We chose to manipulate treatment package using a within-participant design due to practical constraints with the availability of students. This way, all students in all classes received each treatment package, and we avoided assigning treatment packages to individual classes for which students could not be randomly assigned. However, this could be a limitation of the reported experiment because carryover effects may have occurred between treatment packages. For instance, experiencing both overt and covert retrieval could encourage better covert retrieval. Even so, this would reduce the magnitude of our obtained effects suggesting that a between-participant design would likely reveal larger effects. In other words, the impact of treatment package on adolescents’ learning may be more substantial if they only get to experience one treatment eliminating the possibility of strategy carry-over.

A limitation inherent to covert retrieval is that it is difficult to establish what students are doing when mentally retrieving information. This same limitation exists for restudy comparisons that do not require students to copy restudied information. However, this issue does not arise for overt retrieval because an objective retrieval response is recorded. These are ongoing challenges for the field. Identifying methodology to address these concerns will be important for refining theory on student learning. One avenue could be to include subjective assessments for students to report the degree to which they tried to retrieve the definition or strategies they used during covert retrieval. Another avenue could be to include a no study control comparison condition in future research to establish the degree to which all treatment packages impact learning relative to doing nothing. This approach would be less useful from an applied perspective, but it would be valuable for establishing the degree to which covert retrieval enhances learning relative to not studying, which provides insight into students’ willingness or ability to mentally retrieval information. Another possibility is that feedback may be critical for students’ covert retrieval activity. We gave students feedback in all treatment packages to avoid introducing a between-condition confound and, due to the applied nature of this work in authentic middle school classrooms, we prioritized conditions that would be most likely for enhancing student learning (e.g., Bangert-Drowns et al., 1991; Pashler et al., 2005). However, an important path forward for laboratory research will be to investigate the effectiveness of covert retrieval when students learn complex concepts with or without feedback. It is possible that students began to anticipate feedback on covert retrieval trials, which may have led some to disengage in covert retrieval and simply wait for the feedback. This may make covert retrieval more similar to a restudy opportunity in the context of receiving feedback during learning.

## 5. Conclusions

In conclusion, overt retrieval practice best supported adolescents’ learning of concepts in their middle school science classes. Covert retrieval and restudy were typically less effective for students’ long-term learning, although we did find some differences between terms and test types. We recommend that teachers and students use overt retrieval when time is available and the stakes for correct recall are high. Additional research should identify methods to increase the effectiveness of covert retrieval for increasing adolescents’ learning.

## Figures and Tables

**Table 1:** Number of students in each counterbalanced condition during the learning sessions (i.e., Sessions 1–5). O = Overt; R = Restudy; C = Covert.

	Treatment Package Order					
Session	O, R, C	O, C, R	R, O, C	R, C, O	C, O, R	C, R, O
1	12	18	20	0	0	15
2	0	12	0	15	18	20
3	15	0	12	18	20	0
4	20	0	18	12	15	0
5	0	15	0	20	12	18
Total	47	45	50	65	65	53

**Table 2:** Number of students who learned via each treatment package for each science term. * number of students reflects experimenter error in procedure.

	Treatment Package Type		
Term	Overt	Covert	Restudy
Cold Front	35	18	12
Convection	18	32	15
Convection Current	20	18	27
Coriolis Effect	18	32	15
Deep Current	32	18	15
Global Winds	18	27	20
Jet Stream	27	20	18
Local Winds	18	35	12
Meteorology	0 *	15	50 *
Ocean Current	32	15	18
Surface Current	15	32	18
Upwelling	30	15	20
Warm Front	32	18	15
Weather	15	12	38
Weather Map	15	18	32

**Table 3:** Mean percent correct on the final short-answer test for each science term and treatment package. Standard errors are provided in parentheses. Terms are presented with the number of idea units for each. Due to experimenter error, the term meteorology was not learned using the overt retrieval treatment package. * significant impact of treatment package + non-significant trend of treatment package.

		Treatment Package Type			
Term	Idea Units	Overt	Covert	Restudy	Overall
Cold Front	4	42.86 (4.98)	35.29 (7.29)	52.08 (10.64)	42.58 (3.88)
Convection	3	11.76 (3.12)	8.85 (2.59)	8.89 (2.75)	9.64 (1.65)
Convection Current	3	13.33 (3.75)	9.80 (3.52)	17.28 (4.02)	14.06 (2.26)
Coriolis Effect +	4	15.28 (3.58)	5.08 (2.62)	8.33 (2.64)	8.65 (1.80)
Deep Current *	3	58.85 (6.03)	37.25 (6.31)	43.33 (6.04)	49.48 (3.87)
Global Winds	1	52.78 (8.55)	62.96 (5.72)	42.50 (7.50)	53.85 (4.15)
Jet Stream +	2	40.74 (7.45)	18.75 (6.25)	20.83 (7.34)	28.46 (4.31)
Local Winds	2	13.24 (3.79)	7.14 (2.19)	16.67 (5.62)	10.55 (1.91)
Meteorology	3		47.78 (8.74)	38.67 (4.22)	40.77 (3.82)
Ocean Current	2	76.56 (5.01)	66.67 (7.97)	65.28 (8.37)	71.15 (3.85)
Surface Current	3	32.22 (7.71)	24.48 (3.89)	16.67 (5.35)	24.22 (3.4)
Upwelling	3	23.56 (6.22)	34.44 (10.72)	8.33 (5.05)	21.35 (4.21)
Warm Front	4	42.97 (5.83)	42.65 (9.10)	50.00 (8.80)	44.53 (4.26)
Weather	5	47.33 (5.11)	34.17 (7.33)	23.51 (4.03)	31.09 (3.17)
Weather Map	5	32.67 (5.97)	21.76 (5.51)	23.13 (3.40)	25.00 (2.65)

**Table 4:** Mean percent correct on the multiple-choice test for each science term and treatment package. Standard errors are provided in parentheses. Terms are presented with the number of idea units for each. Due to experimenter error, the term meteorology was not learned using the overt retrieval treatment package. * significant impact of treatment package + non-significant trend of treatment package.

		Treatment Package Type			
Term	Idea Units	Overt	Covert	Restudy	Overall
Cold Front	4	65.71 (8.14)	66.67 (11.43)	75.00 (13.06)	67.69 (5.85)
Convection	3	44.44 (12.05)	31.25 (8.32)	46.67 (13.33)	38.46 (6.08)
Convection Current	3	25.00 (9.93)	33.33 (11.43)	29.63 (8.96)	29.23 (5.69)
Coriolis Effect *	4	61.11 (11.82)	25.00 (7.78)	60.00 (13.09)	43.08 (6.19)
Deep Current	3	65.63 (8.53)	61.11 (11.82)	86.67 (9.09)	69.23 (5.77)
Global Winds +	1	44.44 (12.05)	55.56 (9.75)	20.00 (9.18)	41.54 (6.16)
Jet Stream	2	74.07 (8.59)	55.00 (11.41)	66.67 (11.43)	66.15 (5.91)
Local Winds	2	44.44 (12.05)	34.29 (8.14)	58.33 (14.86)	41.54 (6.16)
Meteorology	3		80.00 (10.69)	62.00 (6.93)	66.15 (5.91)
Ocean Current +	2	83.33 (11.24)	48.57 (8.57)	72.22 (10.86)	61.54 (6.08)
Surface Current	3	66.67 (12.60)	43.75 (8.91)	50.00 (12.13)	50.77 (6.25)
Upwelling	3	43.33 (9.20)	46.67 (13.33)	20.00 (9.18)	36.92 (6.03)
Warm Front	4	71.89 (8.08)	55.56 (12.05)	66.67 (12.60)	66.15 (5.91)
Weather *	5	73.33 (11.82)	91.67 (8.33)	42.11 (8.12)	58.46 (6.16)
Weather Map +	5	100 (00)	72.22 (10.86)	68.75 (8.32)	76.92 (5.27)

## Data Availability

All materials, raw data, and class-level analyses have been uploaded to the Open Science Framework and can be freely accessed at https://osf.io/p4cq8 (accessed on 16 June 2025). The study’s design and analyses were not pre-registered.
